# Prevalence of Hypertension in a Tribal Land Locked Population at High Altitude

**DOI:** 10.1155/2016/3589720

**Published:** 2016-02-17

**Authors:** Sunil Kumar Raina, Vishav Chander, Chaman Lal Prasher, Sujeet Raina

**Affiliations:** ^1^Community Medicine, Dr. Rajendra Prasad Government Medical College, Tanda 176001, India; ^2^Internal Medicine, Dr. Rajendra Prasad Government Medical College, Tanda 176001, India

## Abstract

*Introduction.* Extensive pubmed search reveals paucity of data on prevalence of hypertension in tribal population at high altitude. The data is all the more scarce from our part of India. Studies among tribal populations at high altitudes provide an interesting epidemiological window to study human evolution and adaptation to hypobaric hypoxia.* Material and Methods*. 401 participants above the age of 20 years were evaluated for blood pressure using a stratified simple random technique among villages located at high altitude.* Results*. Out of a total of 401 individuals studied 43 (males: 35; females: 8) were identified as hypertensive yielding a crude prevalence of 10.7%. The prevalence was higher in males (35/270; 12.9%) as compared to females (8/131; 6%). Prevalence was the highest in the age group of 30–39 among males (16/35; 45.7%) while it was the highest in the age group of 40–49 among females (7/8; 87%).* Conclusions.* Prevalence of 10.5% is noteworthy when interpreted in light of prevalence of hypertension in general population especially if hypobaric hypoxia is considered to have a protective effect on blood pressure in high altitude native populations.

## 1. Introduction

An extensive pubmed search reveals paucity of data on prevalence of hypertension in tribal population at high altitude. The data is all the more scarce from our part of India. A few studies in this regard reveal contradictory views on the prevalence of hypertension among populations at high altitude. This contradiction in prevalence is even reflected in two studies conducted in Spiti valley (4000 m) of Himachal Pradesh state, although the reason for difference in prevalence at Spiti could partly be attributed to the difference in cut-off used for defining hypertension [1]. Similar to this, studies conducted among high altitude natives of Andes [2–5], Pamirs and Tienshan [6], Amaras region of Ethiopia [7], Sherpas of Nepal [8], Tibetans of India [9], and rural high altitude natives in Greater Himalayas [10] have shown BP values of natives to be lower than the residents of plains and low altitude. However, studies among Tibetans of Lhasa [11], high altitude natives of Saudi Arabia [12], high altitude natives of Ethiopia [13], and high altitude cold zone cattle-breeders of Mongolia [14] have also shown opposite results.

In health and diseases research, tribal populations at high altitude provide an interesting epidemiological window to study human evolution and adaptation to hypobaric hypoxia.

Civilizations with longest history of ancestry (tribal populations living at high altitude) are likely to be genetically more adapted than ones with shorter history of ancestry to natural stressors. It is supposed that the natives of Tibetan plateau of Himalayan region have longer history of ancestry than civilizations in other mountainous regions [15]. The course of evolution and adaptation may have modified many of the human physiological processes, blood pressure being one of them.

## 2. Material & Methods 

### 2.1. Background

Pangi valley is a remote, rugged, and poorly developed tribal area in Himachal Pradesh state. Pangi valley is divided into Saichu, Hudan Bhatori, Chasak Batori, and Sural Bhatori valleys [16]. These are inhabited at elevations of 7,000 feet (2,100 m) to 12,000 feet (4000 m) above sea level. Roads are poor, with few of them surfaced. The entry to the valley is through the Saach Pass. The valley is approachable only between July end and October through Saach Pass, which remains closed by heavy snow at other times of the year.

The Pangi tehsil covers 1,601 square kilometres (618 sq mi) and had a population of 17,598 at the 2001 census. Pangi has 16 panchayats and 54 inhabited villages.

The major tribes inhabiting this area are Pangwals, Lahaula, and Bhot or Bodh. They belong to different castes. The inhabitants of Pangi originally seem to have converged on Pangi from various quarters but the seclusion and inaccessibility forced by geography have wedded these castes into a single tribal community. The villages of Pangi have seen least of in-migration and are almost entirely inhabited by local tribals [16].

The staple food in Pangi is barley, elo (rye), wheat, buckwheat, suil, and chana (both inferior kinds of millets). The wheat and rice are supplied by the government. Further certain grasses and roots like Kangash and Chukri are also consumed. Mutton is often eaten in the winters and on special occasions. Potato is also an important component of diet [16].

Barley, elo, phullan, and bres (buckwheat) and to a small extent wheat are ground into meal for bread which is baked into various forms. Moreover, suil, elo, and barely are parched into ground into flour, called* Sattu*, and eaten without cooking either with buttermilk, water, or tea. Walnut oil and ghee are used mainly for frying vegetables and pulses. People take meals thrice a day. Villagers in Pangi use liquor extensively. This liquor is mostly brewed locally. In addition to liquor, tea, prepared from tea leaves purchased from market and Choga (tea made from local plant species), is also consumed. Choga is prepared by boiling water, milk, salt, and Choga leaves [16].

### 2.2. Methods

#### 2.2.1. Study Participants

Assuming a prevalence of 5% with target population of around 5000 from the study area (the high altitude values), required sample size calculated was 377 at 99.9% CI. All individuals aged 20 years and above from Pangi were the target population screened. A stratified simple random technique was used to include participants for this study. All the villages from Sural Batori valley (10,000 feet; 3048 meters) and Chasak Batori valley (12,000 feet; 3658 meters) were stratified to 2 groups of villages. Villages from each group were selected using random number table so that about 250 eligible individuals could be selected from each of the 2 altitude groups. A higher number was chosen to account for nonwillingness of individuals to participate in the study. A total of 401 individuals participated in the study giving us a response rate of 90%.

#### 2.2.2. Study Design

A total of 401 participants aged above 20 years were included in this study.

A two-stage stratified sampling design was used to select a representative sample of the adult population over 20years of age. The occupation was interviewed from all the participants and classified into four groups: agriculturists, government service, private services, shopkeeper/business, retired from service, and others (housewife, manual labourer, monk, and with no job). Only a full-time housewife was regarded as a housewife while a housewife who also worked as an agriculturist was classified as agriculturist. Further each study participant was interviewed to record information pertaining to history of being aware of having hypertension, record of blood pressure, being aware of having diabetes, and history of tobacco and alcohol consumption.

Anthropometric measurements including weight and height were obtained using standard techniques. Height was recorded with the help of a stadiometer to the nearest of 5 mm. Weight was measured by a digital weighing machine to the nearest 100 g and was calibrated using standard weight every day.

The body mass index (BMI) was calculated using the formula, weight (kg)/(height (m))^2^. BMI ≥ 23 kg/m^2^ was defined as overweight. Blood pressure was measured using an automatic device (HEM 7000; OMRON Life Science Co. Ltd., Kyoto, Japan). Blood pressure was measured twice after taking at least a 5 min rest in a sitting position and the mean of systolic blood pressure (SBP) and diastolic blood pressure (DBP) was calculated. SBP ≥ 140 mm Hg and/or DBP of ≥90 mm Hg and/or taking current antihypertensive medicine was defined as hypertension.

## 3. Results

A total of 401 (males: 270; 67.3% and females: 131; 32.7%) participants aged between 20 and 94years were included in the study. Majority (61%) of the participants belonged to age group of 30–49. The details are provided in Table 1. This table also provides us with details on the prevalence of hypertension. Out of a total of 401 individuals studied 43 (male: 35; female 8) were identified as hypertensive yielding a crude prevalence of 10.7%. The prevalence was higher in males (35/270; 12.9%) as compared to females (8/131; 6%). Prevalence was the highest in the age group of 30–39 among males (16/35; 45.7%) while it was the highest in the age group of 40–49 among females (7/8; 87%).

Table 2 shows the characteristics of all variables of the study participants. Smoking is seen to be common in males with more than 50% reporting as current smoker, occasional smoker, or ex-smoker. Interestingly 9.2% of females also were reported as current smoker. Use of alcohol was reported by a majority (69%) of male participants compared to only 0.8% females. The maximum number of participants was illiterate (males: 20%; females: 61.8%).

Table 3 provides details on mean and median of height, weight, body mass Index (BMI), systolic blood pressure (SBP), and diastolic blood pressure (DBP) according to gender of the study participants. The mean and the median BMI for both male and female gender is within normal limits with little difference between the two (males: 20.80 ± 2.73, 20.68; females: 20.41 ± 2.42; 19.56).

The mean and median SBP for males is 124.56 ± 17.44 and 124 while for females it is 115.82 ± 14.03 and 115, respectively. The mean DBP for males is 76.68 ± 10.45 and 76 while for females it is 73.31 ± 8.83 and 73, respectively. The mean and median population SBP for residents of Pangi is 121 ± 16.89 and 120, respectively. The mean and median population DBP for residents of Pangi is 75.58 ± 10.07 and 75, respectively.

Table 4 reports on awareness of study participants about their status of blood pressure and blood sugar. A large proportion of males (215/270: 80%) and females (116/131: 88.5%) reported not having got examined for raised blood pressure in the last 5 years.

**Figure 1:**
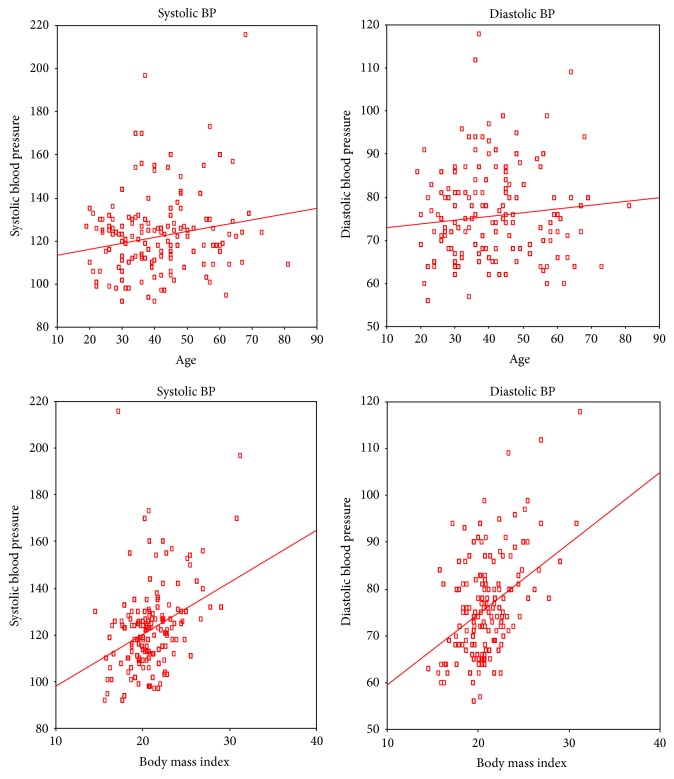
Corelation of blood pressure with age and body mass index (BMI). Systolic BP and age (Pearson correlation: 0.196; significance (2 tailed): ≤0.001). Diastolic BP and age (Pearson correlation: 0.105; significance (2 tailed): 0.035). Systolic BP and BMI (Pearson correlation: 0.349; significance (2 tailed): ≤0.001). Diastolic BP and BMI (Pearson correlation: 0.397; significance (2 tailed): ≤0.001).

**Figure 2:**
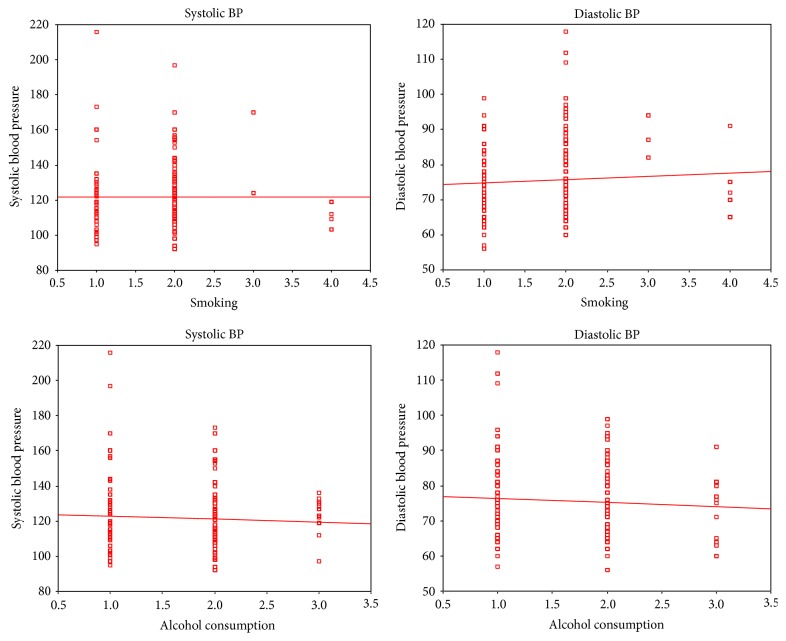
Corelation of blood pressure with smoking and alcohol. Systolic BP and smoking (Pearson correlation: −0.002; significance (2 tailed): 0.966). Diastolic BP and smoking (Pearson correlation: 0.059; significance (2 tailed): 0.240). Systolic BP and alcohol consumption (Pearson correlation: −0.062; significance (2 tailed): 0.218). Diastolic BP and alcohol consumption (Pearson correlation: −0.068; significance (2 tailed): 0.172).

**Figure 3:**
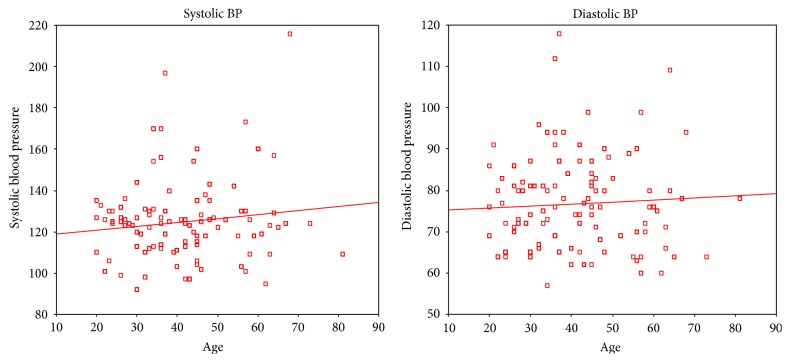
Corelation of blood pressure with age among male participants. Systolic BP and age (Pearson correlation: 0.138; significance (2 tailed): 0.023). Diastolic BP and age (Pearson correlation: 0.059: significance (2 tailed): 0.333).

**Figure 4:**
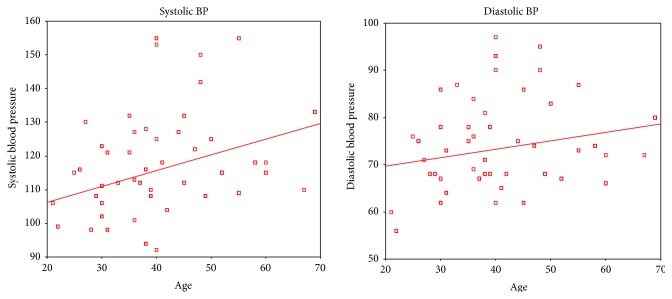
Corelation of blood pressure with age among female participants. Systolic BP and age (Pearson correlation: 0.388; significance (2 tailed): <0.001). Diastolic BP and age (Pearson correlation: 0.238; significance (2 tailed): 0.006).

Figure 1 reports on the corelation of SBP and DBP with age and body mass index (BMI). It is observed that as the age increases both SBP and DBP raise. Similarly as the BMI rises both SBP and DBP rise. However, the correlation coefficient was found to be not significant for age and diastolic blood pressure (DBP) pointing to constancy in DBP with age.  Figure 2 reports on the corelation of SBP and DBP with smoking and alcohol. It is observed that SBP and DBP rise with smoking while no such relation is observed with alcohol. However, the correlation coefficients for both smoking and alcohol are not significant.  Figure 3 reports on the corelation of SBP and DBP with age among males. There is no significant increase in blood pressure with age among males. The lack of significance is more predominant in DBP. However, there is significant increase in blood pressure with age among females (Figure 4). The significance is highly appreciable in SBP.

## 4. Discussion

Globally nearly 140 million people reside at high altitude, defined as elevations above 2500 m (8000 ft.) [17]. Though global distribution of people at high altitude is a small fraction in some countries a sizeable proportion of population live and reproduce at high altitude. In Himachal Pradesh, India, 30% of the population resides at high altitude [18].

High altitude environment poses distinct challenges to highlanders and consequent developmental and genetic adaptations are instances of evolutionary modifications required to survive there. Hypoxia is the fundamental adaptive challenge at high altitude and extensive impact on respiratory and cardiovascular system including systemic blood pressure is expected. The extent to which myriad of cardiovascular adaptations affects systemic blood pressure is controversial. Interpretation of information from high altitude residents is influenced by many factors including the altitude of residence, the physical activity patterns of the subjects, and the possible role of genetic changes that may have resulted in advantageous physiological adaptations to a low oxygen environment. There are few population-based studies examining the effects of chronic exposure to high altitude on systemic blood pressure. Earlier studies established that people who reside at higher altitudes for several years apparently have a decrease in both systemic systolic and diastolic pressure [19, 20]. However, various studies on prevalence of hypertension among natives at high altitudes had conflicting results [2–14]. A study done in Andean highlanders living at 4300 m above the sea level showed a low prevalence of high blood pressure [5]. However, more recent studies done in Tibetan and Andean highlanders suggest that the prevalence of hypertension is similar or higher than in people living at the sea level [9, 14].

The present population-based cross-sectional study was conducted to determine the prevalence of hypertension in a tribal land locked population at high altitude. The study population belongs to an ethnically homogenous population of natives of Pangi valley residing at an altitude of 3048 meters to 3658 meters.

Analysis of the results revealed that overall prevalence of hypertension was 10.7%. The prevalence of hypertension at 10.7% is much lower than the prevalence reported (36%) for mainland Himachal Pradesh [21]. The prevalence is also lower than the one reported (22.5%) in the recent past by other investigators from high altitude Himachal Pradesh [1]. However, prevalence is higher than one earlier study at high altitude populations which reported a prevalence of only 2% [10]. Although this could be attributed to the cut-off of 160/95 mm of Hg to diagnose hypertension used in the earlier study. Therefore, it is likely that this has underestimated the true prevalence of hypertension in high altitude natives, which could otherwise have been significant.

Importantly there is no correlation between advancing age and diastolic blood pressure [10]. This constancy of blood pressure with age has also been reported from Easter Island [22] in a community living the traditional way of life. However, systolic blood pressure seems to rise with advancing age. The rise in systolic blood pressure with age is more appreciable in female than male participants.

The prevalence of obesity and diabetes is less in the higher altitude population; this suggests the likely differences in the level of physical activity which is also substantiated by the fact that group 2 is constituted by more farmers. The higher physical activity index may have protective effect independent of BMI which needs to be explored before concluding the protective effects of hypobaric hypoxia induced vascular adapted responses as a mechanism for lower prevalence of hypertension. There appears to be a dichotomy, in which hypertension at lower altitudes is associated more with obesity and classical cardiovascular risk factors, whereas at high altitude the frequency of hypertension correlates more with the presence of polycythemia and hyperuricemia [23]. Studies from different parts of world, conducted in different populations have also shown association of urbanisation, increase in age, socioeconomic status, waist circumference, and BMI with increase in the prevalence of hypertension [24–30].

## 5. Conclusions

The overall prevalence of 10.5% is noteworthy when interpreted in light of prevalence of hypertension in general population especially if hypobaric hypoxia is considered to have a protective effect on blood pressure in high altitude native populations.

## Figures and Tables

**Table 1:** Demographic profile of study participants and prevalence of hypertension among study participants.

Age group (years)	Number (%)			Mean age (SD)			Hypertensive *n* (%)			Nonhypertensive *n* (%)		
	Male	Female	Total	Male	Female	Total	Male	Female	Total	Male	Female	Total
20–29	56 (14.0)	17 (4.2)	73 (18.2)	24.89 (2.77)	25.76 (2.61)	25.10 (2.74)	1 (0.2)	—	1 (0.2)	55 (13.7)	17 (2.61)	72 (18.0)
30–39	82 (20.4)	60 (15.0)	142 (35.4)	33.66 (3.03)	34.45 (3.72)	33.99 (3.35)	16 (4.0)	—	16 (4.0)	66 (16.5)	60 (15.0)	126 (31.4)
40–49	76 (19.0)	27 (6.7)	103 (25.7)	44.16 (2.45)	44.37 (3.34)	44.21 (2.70)	8 (2.0)	7 (1.7)	15 (3.7)	68 (17.0)	20 (5.0)	88 (21.9)
50–59	30 (7.5)	16 (4.0)	46 (11.5)	55.50 (2.56)	53.69 (2.82)	54.87 (2.76)	3 (0.7)	1 (0.2)	4 (1.0)	27 (6.7)	15 (3.7)	42 (10.5)
≥60	26 (6.5)	11 (2.7)	37 (9.2)	64.54 (5.83)	65.55 (4.43)	64.84 (5.41)	7 (1.7)	—	7 (1.7)	19 (4.7)	11 (2.7)	30 (7.5)

Total	270 (67.3)	131 (32.7)	401 (100.0)	40.19 (12.64)	40.33 (11.60)	40.23 (12.30)	35 (8.7)	8 (2.0)	43 (10.7)	235 (58.6)	123 (30.7)	358 (89.3)

**Table 2:** Frequency distribution of different variables among study participants.

Characteristic	Male *n* (%)	Female *n* (%)	Mean systolic BP ± SD	Mean diastolic BP ± SD
Smoking habits				
Smoker	115 (42.6)	12 (9.2)	121.08 ± 17.47	74.03 ± 9.61
Occasional smoker	7 (2.6)	—	143.71 ± 24.58	88.57 ± 5.47
Ex-smoker	15 (5.5)	—	113.60 ± 7.25	71.20 ± 6.80
Nonsmoker	133 (42.3)	119 (90.8)	121.89 ± 16.33	76.26 ± 10.21
Alcohol consumption				
Alcoholic	159 (58.9)	1 (0.8)	123.62 ± 18.13	76.59 ± 10.77
Occasional alcoholic	26 (9.6)	—	123.96 ± 7.72	75.50 ± 8.92
Nonalcoholic	85 (31.5)	130 (99.2)	120.01 ± 16.62	74.84 ± 9.63
Educational status				
Illiterate	54 (20.0)	81 (61.8)	119.71 ± 19.28	73.33 ± 7.65
Primary school	34 (12.6)	15 (11.4)	110.02 ± 13.69	68.55 ± 8.24
High school not completed	50 (18.5)	10 (7.6)	121.13 ± 11.12	75.10 ± 9.26
High school	32 (11.8)	1 (0.8)	124.67 ± 11.05	77.97 ± 9.12
Secondary school	43 (15.9)	10 (7.6)	125.57 ± 12.27	81.55 ± 10.94
Graduate	18 (6.7)	—	123.56 ± 12.99	79.61 ± 9.58
Postgraduate/professional degree	24 (8.9)	14 (10.8)	137.68 ± 19.63	84.55 ± 10.46
Others	15 (5.6)	—	117.27 ± 7.52	66.73 ± 3.39
Occupation				
Agriculture	119 (44.1)	46 (35.1)	122.22 ± 17.26	75.33 ± 9.31
Government service	77 (28.5)	23 (17.5)	124.95 ± 17.82	77.80 ± 10.86
Private service	9 (3.3)	6 (4.6)	130.60 ± 18.29	83.53 ± 13.53
Shopkeeper/business	20 (7.4)	12 (9.2)	118.22 ± 13.33	73.84 ± 9.19
Retired	3 (1.1)	—	116.33 ± 16.77	68.67 ± 6.35
Others	42 (15.6)	44 (33.6)	116.87 ± 14.69	72.97 ± 9.14

**Table 3:** Anthropometric characteristics of study participants.

	Mean ± SD	Median	95% confidence interval
Height (meters)			
Male	1.65 ± 0.05	1.65	1.64–1.65
Female	1.57 ± 0.04	1.57	1.56–1.57
Total	1.62 ± 0.06	1.62	1.62–1.63
Weight (kilograms)			
Male	56.26 ± 7.51	56.00	55.36–57.16
Female	50.32 ± 7.02	48.60	49.10–51.53
Total	54.32 ± 7.86	53.00	53.55–55.09
Body mass index (kilograms/m^2^)			
Male	20.80 ± 2.73	20.68	20.47–21.13
Female	20.41 ± 2.42	19.56	19.99–20.83
Total	20.67 ± 2.64	20.44	20.41–20.93
Systolic blood pressure (mm of Hg)			
Male	124.56 ± 17.44	124.00	122.47–126.65
Female	115.82 ± 14.03	115.00	113.39–118.24
Total	121.71 ± 16.89	120.00	120.05–123.36
Diastolic blood pressure (mm of Hg)			
Male	76.68 ± 10.45	76.00	75.43–77.93
Female	73.31 ± 8.83	73.00	71.79–74.84
Total	75.58 ± 10.07	75.00	74.59–76.57

**Table 4:** Awareness regarding blood pressure among study participants.

Question	Number (%)		
	Male	Female	Total
When was your blood pressure last measured?			
Within past 12 months	34 (8.5)	25 (6.2)	59 (14.7)
1–5 years	21 (5.2)	15 (3.7)	36 (9.0)
Not within past 5 years	215 (67.3)	91 (22.7)	306 (76.3)
Do you know that your blood pressure is raised or not?			
Yes	9 (2.2)	8 (2.0)	17 (4.2)
No	256 (63.8)	116 (28.9)	372 (92.8)
Uncertain	5 (1.2)	7 (1.7)	12 (3.0)
Are you currently taking antihypertensive drugs?			
Yes	7 (1.7)	4 (1.0)	11 (2.7)
No	263 (65.6)	127 (31.7)	390 (97.3)
Do you know that you have diabetes?			
Yes	5 (1.2)	—	5 (1.2)
No	258 (64.3)	128 (31.9)	386 (96.3)
Uncertain	7 (1.7)	3 (0.7)	10 (2.5)
